# In Vitro Effects of Combining Resin Infiltration and At‐Home Bleaching on Hydrogen Peroxide Penetration, Color Change and Enamel Morphology

**DOI:** 10.1111/jerd.70190

**Published:** 2026-05-19

**Authors:** Bruno Baracco, Taynara S. Carneiro, Michel Wendlinger, Michael W. Favoreto, Laura Ceballos, Alessandro D. Loguercio

**Affiliations:** ^1^ Health Sciences Faculty, IDIBO Research Group Rey Juan Carlos University Alcorcón Madrid Spain; ^2^ Department of Restorative Dentistry, School of Dentistry State University of Ponta Grossa Ponta Grossa Paraná Brazil; ^3^ Department of Restorative Dentistry and Cariology, Adhesive Dentistry Research Group Institute of Dentistry, University of Turku Turku Finland; ^4^ Department of Restorative Dentistry University of São Paulo São Paulo São Paulo Brazil

**Keywords:** bleaching agents, dental enamel permeability, hydrogen peroxide, in vitro techniques, tooth bleaching

## Abstract

**Objective:**

To evaluate the effect of resin infiltration (RI) prior to at‐home bleaching on hydrogen peroxide (HP) penetration into the pulp chamber, color change, and enamel morphology in human teeth with sound enamel or white spot lesions (WSLs), using different RI protocols.

**Materials and Methods:**

Sixty human premolars were randomly allocated into six groups (*n* = 10), according to enamel substrate (sound/WSLs) and treatment: at‐home bleaching with 16% carbamide peroxide only (CP); RI with one prior acid‐etching application followed by at‐home bleaching (RI1/CP); and RI with three prior acid‐etching applications followed by at‐home bleaching (RI3/CP). HP penetration was quantified by UV–Vis spectroscopy. Color change was evaluated using a digital spectrophotometer (ΔE₀₀; WI_D_). Enamel surface morphology was analyzed by SEM (*α* = 0.05).

**Results:**

RI significantly reduced HP penetration into the pulp chamber compared with CP alone, with reductions of over 90%, regardless of enamel substrate or RI protocol. Without RI, artificially induced WSLs showed greater HP diffusion than sound enamel (*p* < 0.05). Color outcomes (WI_D_) were comparable between RI‐treated and sound teeth. SEM showed more homogeneous surfaces after RI.

**Conclusions:**

RI prior to at‐home bleaching with 16% CP reduced HP penetration into the pulp chamber without compromising bleaching efficacy. Comparable color outcomes were achieved in teeth with WSLs, irrespective of prior acid‐etching applications.

**Clinical Significance:**

When combined with at‐home bleaching, RI may reduce HP diffusion into the pulp chamber and improve esthetic integration of artificially induced WSLs through optical masking. However, these findings are based on an in vitro model and should be interpreted with caution regarding clinical outcomes.

## 1 Introduction

Patient demand for esthetic treatments aimed at improving appearance and self‐esteem continues to increase, while clinicians increasingly prioritize minimally invasive approaches [1, 2]. In this context, alterations affecting the color of anterior teeth have become a common source of esthetic concern [3]. The presence of one or multiple white spot lesions (WSLs) on the buccal enamel surface of anterior teeth represents an increasingly common reason for initial consultations [4].

WSLs correspond to hypomineralization defects that may originate before tooth eruption, as observed in fluorosis, molar‐incisor hypomineralization, amelogenesis imperfecta, and traumatic injuries, or may develop after eruption, particularly in early carious lesions [5, 6, 7]. The enamel acquires a whitish and opaque appearance due to subsurface porosities that alter its refractive index and, consequently, its optical properties [8, 9]. Several therapeutic approaches have been described, among which resin infiltration (RI) and dental bleaching stand out as conservative clinical options [10, 11, 12, 13, 14, 15].

RI is widely used in the management of WSLs due to its ability to penetrate and seal enamel microporosities, thereby reducing optical contrast with adjacent sound tissue and improving esthetics [5, 16, 17, 18, 19]. However, the masking effect of RI may vary depending on lesion depth and mineralization, and residual opacity may persist in some cases [20]. In such cases, at‐home dental bleaching, a minimally invasive technique performed with HP (up to 10%) or carbamide peroxide (CP; up to 22%) at lower concentrations using customized trays [21], may be used as a complementary approach to improve color uniformity [10, 11, 16, 22]. Its bleaching effect depends on HP diffusion through dental tissues which, although more controlled than in in‐office bleaching procedures using high‐concentration HP (up to 40%) [23, 24, 25], may still reach the pulp chamber and has been associated with tooth sensitivity [23, 26].

Considering that hypomineralized enamel presents increased porosity, potentially facilitating HP diffusion during bleaching [27, 28], recent evidence has shown that RI significantly reduced the penetration of high‐concentration HP, regardless of substrate (sound enamel or WSL) [27]. This finding suggests a potential protective effect in lower‐concentration protocols; however, this interaction has not yet been investigated in the context of at‐home bleaching.

Also, the efficacy of RI in masking WSLs fundamentally depends on the effective removal of the hypermineralized pseudo intact surface layer, which can be up to 50 μm thick and acts as a barrier to resin penetration into the lesion body [29, 30]. Although a single 120‐s application of 15% hydrochloric acid is the standard protocol, evidence suggests that it removes approximately 30–45 μm of enamel, which may be insufficient for deeper lesions or those with thicker surface layers [30, 31], and, given the difficulty in obtaining and standardizing naturally occurring WSLs for in vitro investigations, the literature supports the use of artificially induced lesions to simulate controlled demineralized enamel conditions [27, 28, 30, 31]. Consequently, clinicians often perform repeated etching cycles to enhance RI and improve esthetic outcomes [32, 33]. However, because excessive etching may result in significant enamel loss and increased surface roughness [34], it is essential to evaluate the impact of different conditioning regimens (e.g., 1 vs. 3 cycles) to establish a protocol that balances optimal masking efficacy with the principles of minimally invasive dentistry [30, 33].

Therefore, the present in vitro study conducted using artificially induced WSLs evaluated HP penetration into the pulp chamber of teeth with different substrates (sound enamel or WSL) subjected to RI performed with different numbers of prior acid‐etching applications (1× or 3×) combined with at‐home bleaching, as well as color change and enamel surface morphology. The research hypotheses to be tested are that: (1) there will be differences in hydrogen peroxide penetration into the pulp chamber; and (2) there will be differences in color change.

## 2 Materials and Methods

### 2.1 Ethical Considerations and Sample Selection Criteria

This in vitro study was submitted for approval by the Research Ethics Committee of the State University of Ponta Grossa (Ponta Grossa, PR, Brazil) and was approved under the agreement number 6.186.109. Sixty healthy human premolars of similar dimensions were obtained from a Human Teeth Bank. The teeth were analyzed under a stereomicroscope at 10× magnification (Lambda LEB‐3, ATTO instruments, Hong Kong, China) to confirm enamel integrity, and specimens presenting morphological changes or cracks were discarded.

Teeth were considered eligible when the combined buccal enamel and dentin thickness ranged from 2.5 to 3.5 mm, as determined by mesiodistal radiographic analysis [35]. Specimens with a Whiteness Index for Dentistry (WI_D_) higher than 20 were excluded, based on measurements obtained using a digital spectrophotometer (VITA Easyshade Advance 4.0, VITA Zahnfabrik, Bad Säckingen, Germany) [36].

### 2.2 Sample Size Calculation

The primary outcome of this study was the quantification of HP within the pulp cavity. Based on a previous study [23], an HP concentration of approximately 0.0508 ± 0.0379 μg/mL was detected in the pulp chamber of teeth subjected to at‐home bleaching with 16% CP. Using a two‐sided test with *α* = 0.05 and 80% power, a minimum of nine teeth in each group was required to detect a difference of 0.1016 μg/mL. An extra tooth was assigned in each group to compensate for possible specimen losses.

### 2.3 Randomization and Allocation

Specimens were first allocated to enamel substrate conditions (sound or WSLs) through a block randomization sequence generated in Microsoft Excel. WSLs were then artificially induced in the selected specimens according to the experimental protocol. Following substrate allocation, a second block randomization procedure was performed to distribute the specimens into the experimental groups. To maintain allocation concealment, the randomization sequences were generated by an independent researcher and implemented using opaque, sealed, and sequentially numbered envelopes, which were opened immediately prior to the experimental procedures. Due to the nature of the interventions, operator blinding could not be performed.

### 2.4 Experimental Groups

Specimens were randomly allocated to six experimental groups (*n* = 10) and stratified according to enamel substrate (sound enamel or WSLs). The experimental groups were defined as follows:

- CP: At‐home bleaching with 16% CP (Whiteness Perfect, FGM Dental Group, Joinville, SC, Brazil) for 14 consecutive days.
- RI1/CP: RI using a single acid‐etching cycle (Icon Smooth Surface, DMG, Hamburg, Germany), followed by a 7‐day interval and subsequent at‐home bleaching with 16% CP for 14 consecutive days.
- RI3/CP: RI using three acid‐etching cycles (Icon Smooth Surface, DMG), followed by a 7‐day interval and subsequent at‐home bleaching with 16% CP for 14 consecutive days.

### 2.5 Preparation of Specimens

The roots were sectioned approximately 3 mm apical to the cementoenamel junction using a low‐speed diamond disk (Isomet 1000, Buehler Ltd., Lake Bluff, USA). Pulp tissue was carefully removed, and the pulp chamber was rinsed with deionized water [35]. Then, access to the pulp chamber was slightly widened using a #1014 round bur (KG Sorensen, Barueri, SP, Brazil), avoiding contact with the buccal inner wall. This enlargement was performed to simplify the subsequent pipetting of 25 μL of solution into the pulp chamber (LABMATE Soft, HTL Lab Solutions, Warsaw, Poland).

To standardize buccal thickness among specimens and to avoid its potential influence on HP penetration, mesiodistal radiographs of each premolar were obtained during the inclusion screening and repeated after preparation (Timex 70C, Gnatus, Ribeirão Preto, SP, Brazil) [35, 37]. Radiographs were taken with the mesial surface in contact with the X‐ray film, using an exposure time of 0.5 s, a 30‐cm focus‐object distance, and settings of 70 kVp–7 mA. The central X‐ray beam was positioned perpendicular (90°) to the distal surface of the tooth. After exposure, digital images were obtained, and buccal thickness was measured using New IDA software (Dabi Atlante, Ribeirão Preto, SP, Brazil). Following specimen preparation, all teeth were maintained in artificial saliva for 1 week prior to the start of the experimental procedures.

### 2.6 Preparation of the Silicone Mounting Rack

To ensure consistent specimen positioning throughout the experimental procedures, a custom rack was designed using heavy‐body silicone (Coltoflax and Perfil Cub Kit, Vigodent, Rio de Janeiro, RJ, Brazil) for each group [27]. The crowns were vertically embedded in the silicone in a predetermined sequence, keeping the pulp chambers accessible. A circular window (6 mm diameter) was later created with a sharp circular‐shaped metal device in the middle third of the buccal surface of each tooth impression. This window exposed a standardized area of vestibular enamel, through which all experimental procedures were performed according to the experimental group [27].

### 2.7 Induction of White Spot Lesions (WSLs)

WSLs were experimentally induced through controlled enamel demineralization using a 14‐day pH‐cycling regimen consisting of alternating demineralization and remineralization periods. Before the cycling process, the entire tooth surface was coated with a waterproof nail varnish, except for a standardized 6‐mm‐diameter circular area on the buccal surface. This exposed region corresponded precisely to the area previously defined on the silicone holder, allowing uniform treatment and preventing solution penetration toward the pulp chamber, thereby minimizing the risk of contamination [27].

Demineralization was achieved using a gel containing 0.1 M lactic acid and 1.5 mM calcium phosphate (CaPO_4_), adjusted to pH 4.5. After each demineralization cycle, specimens were thoroughly rinsed with deionized water and immersed in a remineralizing solution (1.5 mM CaCl_2_, 0.9 mM KH_2_PO_4_, 130 mM KCl, and 20 mM HEPES, pH 7.0) to simulate oral conditions. To ensure chemical stability and effectiveness, the demineralizing medium was renewed after 7 days. At the end of the cycling period, enamel surfaces were inspected to confirm preservation of structural integrity, and the protective nail coating was carefully removed with acetone [38, 39].

### 2.8 Resin Infiltration Protocol

RI was carried out in the RI1/CP and RI3/CP groups, both in sound and WSL specimens, while teeth remained secured in their individualized silicone holders, ensuring standardized exposure of the previously delimited buccal enamel area. The RI system used was Icon Smooth Surface (DMG, Hamburg, Germany), and all procedures were conducted according to the manufacturer's instructions.

Initially, the exposed enamel surface was cleaned with a prophylaxis brush and water and subsequently air‐dried. Surface conditioning was performed using 15% hydrochloric acid gel (Icon Etch; DMG, Hamburg, Germany) applied for 2 min. In the RI1/CP group, the etching procedure was performed once, whereas in the RI3/CP group it was repeated three times following the same protocol. After each etching cycle, the surface was rinsed with water for 30 s and thoroughly air‐dried.

Dehydration was then completed by applying 99% ethanol (Icon Dry; DMG, Hamburg, Germany) for 30 s, followed by air drying. The RI (Icon Infiltrant, composed of TEGDMA and TMPTA; DMG, Hamburg, Germany) was applied in excess to the conditioned enamel and allowed to penetrate for 3 min. Light activation was performed for 40 s using a Valo Grand LED curing unit (Ultradent, South Jordan, UT, USA) operating at 1600 mW/cm^2^ in high‐power mode. A second layer of infiltrant was then applied for 1 min, as recommended by the manufacturer, followed by an additional 40‐s light‐curing cycle. A 7‐day interval between RI and bleaching was adopted to allow resin stabilization, as previous evidence suggests improved stain reduction when this interval is respected [40].

### 2.9 Generation of the Calibration Curve for HP Quantification

All analytical reagents were used as received, without prior purification, and all solutions were prepared with deionized water. A HP standard calibration curve was initially constructed from a 5000 μg/mL stock solution prepared from a concentrated 35% solution (HP, LABSYNTH, Diadema, SP, Brazil). This solution was diluted in acetate buffer (pH = 4) and titrated with potassium permanganate solution to determine its analytical grade and actual HP concentration. Based on the confirmed concentration, serial volumetric dilutions ranging from 0.000 to 0.402 μg/mL were prepared to construct the analytical curve. Known HP concentrations were quantified using a Cary UV–Vis 50 spectrophotometer (Varian, Palo Alto, CA, USA). The calibration curve demonstrated a strong correlation coefficient (*R* = 0.998; data not shown), allowing reliable estimation of HP concentrations in the experimental samples [27, 41].

### 2.10 Bleaching Application and HP Quantification in the Pulp Chamber

Specimens were positioned in the silicone racks, and a 25 μL aliquot of acetate buffer (pH = 4) was placed inside the pulp chamber of each tooth to retain any HP diffusing during the bleaching procedure. A 16% CP gel (Whiteness Perfect, FGM Dental Group, Joinville, SC, Brazil) was applied by a single calibrated operator to the standardized buccal enamel area, following the manufacturer's recommendations (3 h per day). After each bleaching period, the gel was removed with gauze, and the enamel surface was thoroughly rinsed with deionized water. Immediately thereafter, the acetate buffer solution inside the pulp chamber was collected using a mechanical micropipette and transferred to a glass tube. The pulp chamber was subsequently rinsed four times with 25 μL of acetate buffer, and each aliquot was added to the same glass tube to ensure complete recovery of diffused HP.

Thereafter, 2725 μL of distilled water, 100 μL of 0.5 mg/mL (Leucocrystal Violet, Sigma Chemical Co, St Louis, MO, USA) and 50 μL of 1 mg/mL horseradish peroxidase enzyme (Peroxidase Type VIA, Sigma Chemical Co., St. Louis, MO, USA) were added to each tube. This procedure was performed separately for each specimen. The reaction produced a violet‐colored solution with maximum absorbance at 590 nm, which was measured using a Cary 100 UV–Vis spectrophotometer (Varian, Palo Alto, CA, USA), at 590 nm, corresponding to the peak absorbance of the HP‐Leucocrystal Violet reaction. According to Beer–Lambert's law, absorbance is directly proportional to concentration; therefore, HP concentration (μg/mL) in the pulp chamber was determined by comparing the absorbance values with the calibration curve [27, 41]. To simulate the at‐home bleaching treatment (14 days), specimens were daily removed from artificial saliva, rinsed with distilled water, dried, positioned in the silicone racks, submitted to bleaching, rinsed again, and subsequently immersed in freshly prepared artificial saliva at 37°C.

### 2.11 Color Change Evaluation

The initial color of each specimen was assessed under standardized conditions. For sound teeth, baseline color measurements were recorded before any treatment. For specimens with artificially induced WSLs, baseline measurements were obtained immediately after lesion induction and prior to any subsequent procedures. Additional color evaluations were performed after RI (when applicable) and at two time points during the bleaching protocol: after 1 week and after 2 weeks, the latter corresponding to the completion of treatment.

Color parameters (L*, a*, and b*) were measured using a digital spectrophotometer (VITA Easyshade Advance 4.0, Vita Zahnfabrik, Bad Säckingen, Germany). The device tip was positioned through the standardized window in the silicone rack to ensure consistent placement during all measurements. The L* value represents lightness (ranging from 0 = black to 100 = white), a* value indicates to the green‐red axis, and b* value to the blue‐yellow axis [42]. Color change differences between baseline and post‐bleaching was given by the difference between the colors measured with the spectrophotometer using the following time points (after RI, 1 week and 2 weeks) were calculated using the CIEDE2000 formula: Δ*E*₀₀ = [(Δ*L*′/*k*_L_ *S*_L_)^2^ + (Δ*C*′/*k*_C_ *S*_C_)^2^ + (Δ*H*′/*k*_H_ *S*_H_)^2^ + RT (Δ*C*′/*k*_C_ *S*_C_) (Δ*H*′/*k*_H_ *S*_H_)]^1/2^ [43, 44]. For the groups that did not undergo RI (CP), Δ*E*₀₀ values at the “after RI” time point were set to zero, since no color change could be calculated at this stage. This approach was adopted to standardize the evaluation time points and allow comparisons across all experimental groups, despite differences in treatment sequence. The Whiteness Index for Dentistry (WI_D_) was also calculated using the equation WI_D_ = 0.551 × L − 2.324 × a − 1.100 × b [36]. For the CP groups, WI_D_ values at the “after RI” time point corresponded to baseline measurements, as no intervention was performed at this stage. This strategy ensured consistency in data presentation and enabled comparisons across groups.

### 2.12 Enamel Morphology Analysis

After completion of all experimental procedures, three specimens per group were randomly chosen for qualitative surface morphology analysis. The selected teeth were sectioned in the mesiobuccal direction to obtain enamel fragments measuring approximately 5 × 5 mm. These fragments were ultrasonically cleaned to remove surface residues and then stored in a desiccator for 12 h to ensure complete dehydration. Subsequently, the samples were sputter‐coated with a thin gold–palladium layer using a MED 010 sputter coater (Balzers Union, Balzers, Liechtenstein) to provide electrical conductivity. The prepared specimens were examined under a scanning electron microscope (SEM; Balzers Union, Balzers, Liechtenstein). Representative images were captured at 2000× magnification, enabling qualitative analysis assessment of enamel surface morphology and treatment‐related structural changes.

### 2.13 Statistical Analysis

Prior to statistical analysis, data distribution was assessed using the Shapiro–Wilk test, and homogeneity of variances was evaluated using Bartlett's test (data not shown). After confirmation of normal distribution and equal variances, HP concentration detected in the pulp chamber and buccal thickness were analyzed using two‐way ANOVA (treatment × substrate), followed by Tukey's post hoc test. Color change parameters (Δ*E*_00_, and WI_D_) were analyzed by three‐way ANOVA (treatment × substrate × time), followed by Tukey's post hoc test (*α* = 0.05). Effect sizes (Cohen's *d*) were calculated to complement statistical significance. Enamel morphology findings obtained by SEM were subjected exclusively to qualitative descriptive analysis.

## 3 Results

### 3.1 Buccal Surface Thickness and HP Quantification Into the Pulp Chamber

Regarding buccal surface thickness, neither the interaction “treatment” vs. “substrate,” nor the main factors were statistically significant (*p* = 0.84; Table 1). The mean and standard deviation values of the buccal surface thickness for all groups, as determined by radiographs analysis, are described in Table 1.

**TABLE 1:** Mean values (± standard deviations) of the buccal surface thickness and hydrogen peroxide (HP) concentration detected inside the pulp chamber across the experimental groups.

Treatment	Substrate	Buccal thickness (mm; *)	HP (μg/mL; *)
CP	Sound	3.2 ± 0.2 A	0.041 ± 0.014 b
	White spot lesions	3.1 ± 0.2 A	0.076 ± 0.015 c
RI1/CP	Sound	3.2 ± 0.1 A	0.002 ± 0.001 a
	White spot lesions	3.3 ± 0.1 A	0.002 ± 0.000 a
RI3/CP	Sound	3.2 ± 0.1 A	0.003 ± 0.002 a
	White spot lesions	3.2 ± 0.1 A	0.003 ± 0.003 a

*Note:* (*) Identical uppercase letters (buccal thickness) and lowercase letters (HP concentration) within each column indicate statistically similar means (Two‐way ANOVA followed by Tukey's test, *p* > 0.05).

The analysis of HP content within the pulp chamber revealed a significant interaction between treatment and substrate (*p* < 0.001; Table 1). All groups that received RI prior to bleaching showed markedly lower HP penetration into the pulp chamber compared with the CP groups. Specifically, HP levels decreased from 0.041–0.076 μg/mL in CP groups to approximately 0.002–0.003 μg/mL in RI‐treated groups, representing reductions of over 90% (Table 1).

Regarding the substrate factor, within the CP groups, teeth with WSLs exhibited nearly 50% higher HP penetration than sound enamel (0.076 vs. 0.041 μg/mL; Table 1). However, following RI application, HP penetration was similarly low in both substrates, with no significant differences between sound enamel and WSLs groups (RI1/CP; RI3/CP). These findings indicate that RI effectively reduced HP diffusion and eliminated substrate‐related differences (Table 1).

### 3.2 Color Change Evaluation

Statistical analysis revealed no significant three‐way interaction among treatment, substrate, and time factors for Δ*E*_00_ (*p* = 0.77; Table 2). However, all main effects were statistically significant (*p* < 0.001; Table 2). Regarding the treatment factor, no significant differences were observed among groups when analyzed individually, regardless of the evaluation time (Table 2).

**TABLE 2:** Mean values (± standard deviations) of color change (Δ*E*_00_) obtained from objective color assessments in the experimental groups.

Treatment	Substrate	Times		
		After resin infiltration	1 week	2 weeks
CP	Sound	0.0 ± 0.0 E**	7.6 ± 3.5 CD	9.4 ± 2.3 bc
	White spot lesions	0.0 ± 0.0 E**	10.4 ± 1.5 bc	11.6 ± 2.5 AB
RI1/CP	Sound	6.5 ± 1.6 D	6.8 ± 2.3 D	8.4 ± 2.6 CD
	White spot lesions	12.6 ± 3.7 AB	12.7 ± 4.2 AB	13.9 ± 2.3 A
RI3/CP	Sound	5.6 ± 3.6 D	6.0 ± 4.0 D	7.2 ± 3.2 CD
	White spot lesions	10.3 ± 4.3 bc	10.5 ± 4.1 bc	11.8 ± 3.8 AB

*Note:* (*) Identical letters indicate statistically similar values among groups (Three‐way ANOVA followed by Tukey's test, *p* > 0.05). (**) The respective groups did not receive resin infiltration. Because Δ_00_ cannot be calculated from baseline values alone, the color change was considered as 0 for these groups to allow proper statistical analysis among all experimental groups.

In contrast, for the substrate factor, only the CP group did not show a difference between sound enamel and WSLs (Table 2), with a moderate‐to‐large effect size at 2 weeks (Cohen's *d* ≈ 0.9). The application of RI in sound teeth (RI1/CP; RI3/CP) resulted in significantly lower color change compared with the corresponding groups in which RI was applied to WSLs, with large effect sizes observed at baseline (after RI) (RI1/CP: *d* ≈ 2.0; RI3/CP: *d* ≈ 1.4). However, values remained similar to those observed in the CP sound group (~8 Δ*E*_00_ units; Table 2). Conversely, in teeth with WSLs, the presence of RI (RI1/CP; RI3/CP) did not produce significant differences in color change compared with the WSL group without RI (CP), with small effect sizes observed at 2 weeks (*d* < 0.5), despite similar mean values (~12 Δ*E*_00_ units; Table 2).

Regarding WI_D_, no statistically significant interaction was detected among the factors treatment, substrate, and time factors (*p* = 0.95); however, all main effects were statistically significant (*p* < 0.001; Table 3). Baseline WI_D_ values revealed significant differences between substrates in all groups, with substantially lower values observed in the presence of WSL (~7 WI_D_ units; Table 3), corresponding to very large effect sizes (Cohen's *d* > 2.0).

**TABLE 3:** Mean values (± standard deviations) of baseline and post‐bleaching Whiteness Index for Dentistry (WI_D_) measurements across the experimental groups.

Treatment	Substrate	WI_D_ initial (*)	Time points (**)		
			After resin infiltration	1 week	2 weeks
CP	Sound	20.5 ± 5.2 A	20.5 ± 5.2 c	28.6 ± 3.7 b	35.2 ± 2.8 a
	White spot	7.0 ± 6.0 B	7.0 ± 6.0 d	17.7 ± 7.2 c	23.6 ± 6.8 bc
RI1/CP	Sound	20.0 ± 6.0 A	26.3 ± 9.1 ab	28.4 ± 8.6 ab	31.4 ± 8.0 a
	White spot	7.3 ± 6.2 B	27.5 ± 7.8 ab	29.2 ± 5.4 a	31.9 ± 7.3 a
RI3/CP	Sound	21.0 ± 3.5 A	25.2 ± 10.9 abc	28.3 ± 7.2 ab	33.1 ± 5.8 a
	White spot	8.2 ± 4.7 B	26.7 ± 7.4 ab	27.3 ± 6.6 ab	33.1 ± 6.3 a

*Note:* (*) Identical uppercase letters indicate statistically similar values among groups for WI_D_ Initial (One‐way ANOVA followed by Tukey's test, *p* > 0.05). (**) Identical lowercase letters indicate statistically similar values among groups across different time points (Three‐way ANOVA followed by Tukey's test, *p* > 0.05).

Among groups receiving RI (RI1/CP; RI3/CP), no significant differences were observed between substrates throughout the treatment period (Table 3), with negligible to small effect sizes at 2 weeks (*d* < 0.2), indicating a convergence of WI_D_ values between sound enamel and WSLs.

In contrast, in groups without RI (CP), a significant difference between substrates was observed over time, favoring the sound enamel group (Table 3), with moderate‐to‐large effect sizes at 2 weeks (*d* ≈ 1.5). At the end of the treatment, groups treated with RI, regardless of the substrate (RI1/CP; RI3/CP), achieved WI_D_ values (~32 WI_D_ units) comparable to those observed in the sound enamel group treated with CP (~35 units; Table 3), with small effect sizes (*d* < 0.5), indicating similar final color outcomes.

### 3.3 Enamel Morphology Analysis

**FIGURE 1:**
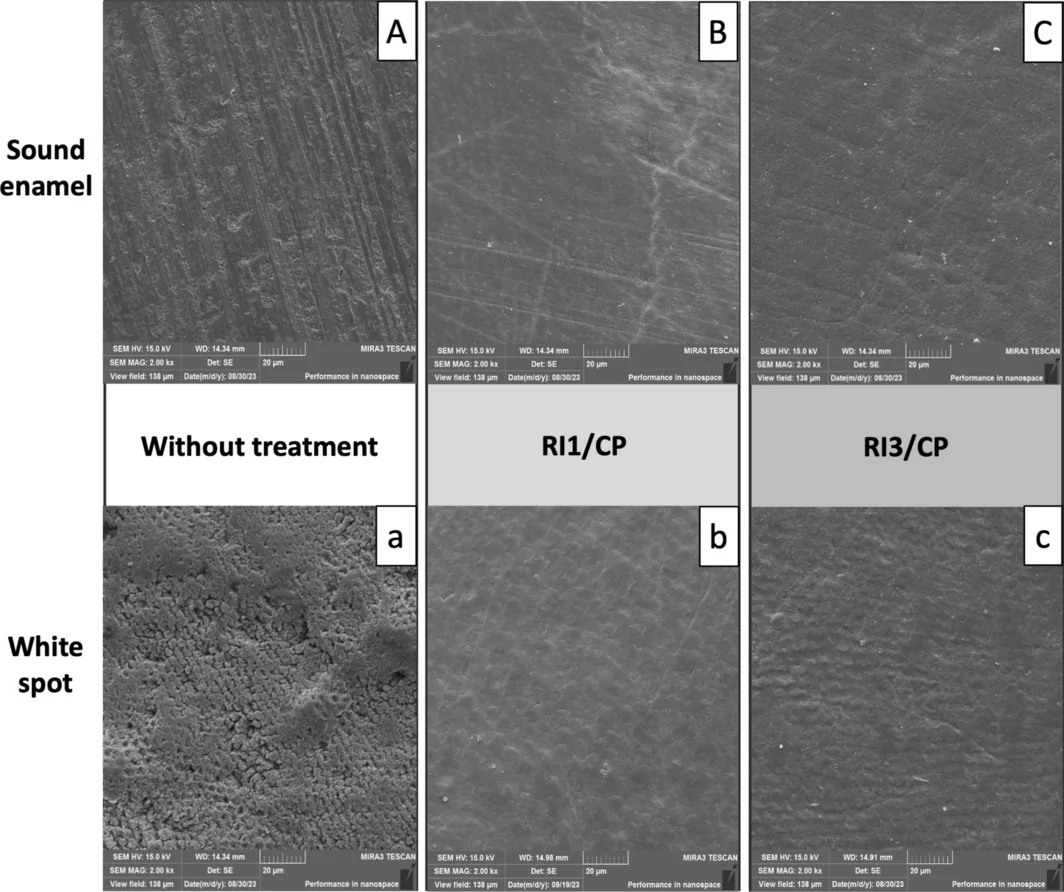
Scanning electron microscopy images comparing sound enamel and enamel with WSL under different treatment conditions. In untreated specimens, sound enamel exhibits a smooth and regular surface morphology (A), whereas the white spot lesion (a) shows a rough, porous, and irregular surface, consistent with deeper surface demineralization. Following RI, both after a single and triple acid‐etching protocol (RI1/CP; RI3/CP), a uniform and homogeneous surface layer is observed on both sound enamel and white spot lesions (B‐b and C‐c). Notably, in treated WSL specimens, previously evident subsurface porosities are no longer visible, suggesting effective resin penetration and infiltration into the demineralized enamel structure.

The WSL exhibits deep and pronounced enamel demineralization compared with sound enamel (Figure 1A‐a). Following RI application, a homogeneous surface layer was observed on both substrates (sound enamel and WSLs), regardless of the protocol used (i.e., RI1/CP or RI3/CP) (Figure 1B‐b,C‐c). In the WSL specimens, the previously demineralized enamel prisms appeared fully infiltrated and filled, indicating effective resin penetration.

## 4 Discussion

The combination of RI with at‐home dental bleaching has been proposed as a conservative alternative for managing WSLs, with the dual objective of enhancing esthetics while preserving dental hard tissues [10, 11]. Despite its increasing clinical application, evidence regarding its impact on HP diffusion, bleaching response, and enamel surface behavior in at‐home protocols has remained limited.

The findings of the present study demonstrated that RI significantly reduced HP penetration into the pulp chamber without compromising bleaching efficacy, regardless of the substrate (sound enamel or WSLs). Furthermore, increasing the number of acid‐etching applications did not result in additional benefits or detrimental effects concerning HP penetration or esthetic performance. Therefore, the research hypotheses were rejected, as significant differences were observed in HP diffusion, and color parameters, among the experimental conditions evaluated.

Considering that buccal enamel thickness may influence HP diffusion toward the pulp chamber [24], this variable was previously standardized through radiographic analysis. No significant effects of thickness were observed on HP penetration or on color‐related parameters. This methodological control supports the interpretation that the differences identified among experimental groups were attributable to the applied interventions and substrate type rather than anatomical variations among specimens [27].

Quantification of HP within the pulp chamber revealed a significant interaction between treatment and substrate. In groups subjected exclusively to bleaching with CP, teeth presenting WSLs exhibited approximately 50% greater HP penetration than sound enamel. This finding aligns with previous evidence associating WSLs with increased enamel permeability, thereby facilitating oxidizing agent diffusion [27, 28]. From a microstructural perspective, WSLs disrupt prism structure and enlarge interprismatic spaces, potentially creating preferential diffusion pathways [6, 7], which may increase HP penetration and could be associated with adverse clinical effects such as tooth sensitivity [25, 28]. However, such outcomes were not directly evaluated in the present study.

In a previous in vitro investigation using in‐office bleaching with 35% HP [27], RI also significantly modified HP diffusion. Following RI application, a marked reduction exceeding 90% in pulpal HP levels was detected in both sound enamel and WSLs, eliminating differences identified between them. This supports the ability of RI to promote surface sealing and reduce the overall permeability. Notably, HP values measured in the RI groups (RI1/CP; RI3/CP) were comparable to those reported for an experimental CP 4% gel (~1.5% HP) [21], with similar diffusion levels (~0.003). This suggests that RI may reduce pulpal HP penetration to levels comparable to lower‐concentration protocols, even when combined with higher‐concentration bleaching agents such as 16% CP.

Although repeated applications of 15% hydrochloric acid are often recommended to facilitate removal of the hypermineralized pseudo intact surface layer and enhance RI in deeper lesions [30, 31, 32, 33], increasing the number of acid‐etching applications (1× vs. 3×) did not result in greater HP penetration. Such an effect might have been anticipated, as more extensive acid conditioning could potentially alter the structural properties of dental tissues [34]; however, the protective benefit of RI appears to be independent of incremental increases in RI depth. While multiple etching cycles may be justified to optimize masking in specific clinical scenarios [32, 33], taken together, these findings suggest that RI acts as a modulator of enamel permeability, equalizing HP diffusion between sound enamel and WSLs.

Color assessment in dental bleaching studies has progressively evolved, with increasing emphasis on metrics that better reflect human visual perception. In the present study, the ΔE₀₀ formula was adopted as one of the analytical parameters [43]. Although statistically significant differences were observed among certain experimental conditions, RI did not appear to compromise bleaching efficacy, regardless of the substrate. However, numerically higher Δ*E*₀₀ values were observed in the WSL groups, which warrants cautious interpretation.

In the literature, when the individual coordinates of the CIELAB system [42], are analyzed (L*, a*, and b*), both sound teeth and those presenting WSLs undergoing bleaching typically demonstrate an increase in L* (lightness), a reduction in a* (green–red axis), and a decrease in b* (blue–yellow axis) [28]. The main difference between substrates lies in their baseline values, as WSLs exhibit significantly lower initial parameters [28], possibly due to alterations in enamel prism structure characteristic of this condition [6, 7]. Although some coordinates in WSLs may approximate those observed in sound teeth after bleaching [28], they generally do not surpass them. In this context, interpretation of the results may be better represented by the WI_D_, which was specifically developed to quantify the perception of dental whiteness. As a more recent parameter focused on whiteness assessment, WI_D_ has been considered methodologically more robust and less prone to distortion compared with previously used formulas [36].

In the WI_D_ analysis, although baseline differences between substrates were expected, by the end of treatment the groups that underwent RI exhibited bleaching values comparable to those observed in sound enamel bleached without RI. A similar outcome was previously in an in‐office bleaching model [27]. Notably, in teeth presenting WSLs, RI appeared to enhance optical integration without compromising bleaching efficacy. By modifying light transmission and scattering within the porous enamel structure, the RI likely reduced visual contrast between the affected areas and the adjacent enamel, thereby promoting a more homogeneous post‐bleaching appearance [5].

Morphological analysis revealed marked structural disorganization in untreated WSLs. Following RI, a more uniform surface and a reduction of porosities were observed, regardless of the number of acid etching applications performed. These findings are consistent with previous studies reporting reduced surface roughness, increased microhardness, and improved bond strength after RI [17, 18, 19, 27], thereby further contributing to the existing body of literature, particularly considering that bleaching procedures performed directly on WSLs often raise concerns regarding enamel structural integrity.

In this context, RI appears to fulfill a dual role: providing optical masking of the lesion while simultaneously modulating substrate permeability. The consistency of these results across both at‐home and in‐office bleaching [27] protocols suggests that RI may be a promising adjunctive approach within minimally invasive esthetic dentistry. Nevertheless, these results should be interpreted within the limitations of an in vitro model, and further clinical studies are required to confirm their biological and clinical implications.

As an in vitro investigation, the findings should be interpreted considering the inherent limitations of the experimental model. Clinical conditions were not fully replicated, as factors such as pulpal pressure, salivary dynamics, and intraoral temperature were not simulated. Additionally, artificially induced WSLs may not fully reproduce the structural complexity of naturally occurring lesions. Another limitation is that the bleaching agent was applied only to the area corresponding to the WSLs, which does not fully reflect conventional at‐home bleaching protocols. Therefore, further research, including well‐designed randomized clinical trials, is necessary to validate these results and to assess the long‐term stability of the observed effects, particularly with regard to tooth sensitivity. Within these limitations, the present data support RI as a predictable and conservative adjunct in the management of WSLs associated with at‐home bleaching, consistent with contemporary principles of tissue preservation.

## 5 Conclusions

Despite the evaluation of different resin infiltration protocols and enamel conditions, the following conclusions can be drawn:

1. Resin infiltration performed prior to at‐home bleaching with 16% carbamide peroxide reduced hydrogen peroxide penetration into the pulp chamber by more than 90% without compromising bleaching efficacy.
2. In teeth with artificially induced white spot lesions treated with resin infiltration, the final shade achieved was comparable to that of sound enamel, with improved color uniformity.
3. Resin infiltration resulted in a more homogeneous and less porous enamel surface compared to untreated white spot lesions, with no significant differences observed between the different acid‐etching protocols.

## Conflicts of Interest

Alessandro D. Loguercio is an Associate Editor of the Journal Esthetic and Restorative Dentistry. In accordance with the journal’s editorial policies, they had no involvement in the peer review of this article and had no access to information regarding its peer‐review process. Full responsibility for the editorial handling of this manuscript was delegated to another editor. The other authors declare no conflicts of interest.

## Data Availability

The data that support the findings of this study are available from the corresponding author upon reasonable request.
